# CH_4_ oxidation in a boreal lake during the development of hypolimnetic hypoxia

**DOI:** 10.1007/s00027-019-0690-8

**Published:** 2019-12-28

**Authors:** Taija Saarela, Antti J. Rissanen, Anne Ojala, Jukka Pumpanen, Sanni L. Aalto, Marja Tiirola, Timo Vesala, Helena Jäntti

**Affiliations:** 1 Department of Environmental and Biological Sciences, University of Eastern Finland, Yliopistonranta 1 E, 70210 Kuopio, Finland; 2 Faculty of Engineering and Natural Sciences, Tampere University, Korkeakoulunkatu 6, 33720 Tampere, Finland; 3 Ecosystems and Environment Research Programme, Faculty of Biological and Environmental Sciences, University of Helsinki, Viikinkaari 1, 00014 Helsinki, Finland; 4 Institute of Atmospheric and Earth System Research (INAR)/Forest Sciences, Faculty of Agriculture and Forestry, University of Helsinki, Viikinkaari 1, 00014 Helsinki, Finland; 5 Helsinki Institute of Sustainability Science (HELSUS), Faculty of Biological and Environmental Sciences, University of Helsinki, Viikinkaari 1, 00014 Helsinki, Finland; 6 Department of Biological and Environmental Sciences, University of Jyväskylä, Survontie 9 C, 40014 Jyväskylä, Finland; 7 Institute of Atmospheric and Earth System Research (INAR)/Physics, Faculty of Sciences, University of Helsinki, Gustaf Hällströmin katu 2, 00560 Helsinki, Finland

**Keywords:** Boreal lake, Greenhouse gases, Hypoxia, Methane, Oxidation, Stable isotopes, Stratification

## Abstract

Freshwater ecosystems represent a significant natural source of methane (CH_4_). CH_4_ produced through anaerobic decomposition of organic matter (OM) in lake sediment and water column can be either oxidized to carbon dioxide (CO_2_) by methanotrophic microbes or emitted to the atmosphere. While the role of CH_4_ oxidation as a CH_4_ sink is widely accepted, neither the magnitude nor the drivers behind CH_4_ oxidation are well constrained. In this study, we aimed to gain more specific insight into CH_4_ oxidation in the water column of a seasonally stratified, typical boreal lake, particularly under hypoxic conditions. We used ^13^CH_4_ incubations to determine the active CH_4_ oxidation sites and the potential CH_4_ oxidation rates in the water column, and we measured environmental variables that could explain CH_4_ oxidation in the water column. During hypolimnetic hypoxia, 91% of available CH_4_ was oxidized in the active CH_4_ oxidation zone, where the potential CH_4_ oxidation rates gradually increased from the oxycline to the hypolimnion. Our results showed that in warm springs, which become more frequent, early thermal stratification with cold well-oxygenated hypolimnion delays the period of hypolimnetic hypoxia and limits CH_4_ production. Thus, the delayed development of hypolimnetic hypoxia may partially counteract the expected increase in the lacustrine CH_4_ emissions caused by the increasing organic carbon load from forested catchments.

**Electronic supplementary material:**

The online version of this article (10.1007/s00027-019-0690-8) contains supplementary material, which is available to authorized users.

## Introduction

Freshwater ecosystems cover 3.7% of the Earth’s non-glaciated land area (Verpoorter et al. 2014), and they are one of the largest natural sources of the global greenhouse gas (GHG) methane (CH_4_) (Bastviken et al. 2011). Approximately half of the lake surface area is located at northern latitudes (Wik et al. 2016), where small lakes in particular tend to have high CH_4_ emissions per unit area (Juutinen et al. 2009). Processes producing GHGs in lakes are connected to their proximate terrestrial environments, because lakes receive terrestrially fixed carbon (C) and emit part of it back to the atmosphere as CH_4_ and carbon dioxide (CO_2_) (Algesten et al. 2003). These processes are especially pronounced in boreal lakes with high loads of dissolved organic matter (DOM) from forested, peat-dominated catchment areas (Kortelainen 1993). Recent studies have shown an increasing trend in the lake and stream water dissolved organic C (DOC) concentrations throughout the boreal zone (Sarkkola et al. 2009; Couture et al. 2012; Pumpanen et al. 2014). This increase is mainly driven by changes in hydrometeorology, i.e. precipitation and air temperature (Sarkkola et al. 2009; Pumpanen et al. 2014); thus, the significance of terrestrial organic C load to aquatic ecosystems might further increase under a changing climate.

In freshwater lakes, dissolved oxygen (DO) depletion due to the decomposition of organic matter (OM) creates suitable redox conditions for methanogenesis, in which CH_4_ is the final product of anaerobic OM decomposition in the absence of alternative electron acceptors (EAs), e.g. nitrate (NO_3_^−^), sulphate (SO_4_^2−^) and iron (Fe^3+^) (Capone and Kiene 1988). However, several studies have also reported methanogenesis in oxic freshwaters (Schulz et al. 2001; Bogard et al. 2014). Once formed in lake sediment or water column, CH_4_ can be either oxidized to CO_2_ by methane-oxidizing microbes, assimilated to biomass, or released to the atmosphere (Kuivila et al. 1988; Bastviken et al. 2002; Kankaala et al. 2006; Wik et al. 2016). The production and oxidation of CH_4_ are controlled by different environmental factors, such as temperature and the availability of oxygen (O_2_), nutrients and OM (Zeikus and Winfrey 1976; Juutinen et al. 2009; Duc et al. 2010; Borrel et al. 2011; West et al. 2016). Besides the production-oxidation processes, it is important to understand CH_4_ transport from the sediment to the atmosphere by diffusion and/or ebullition (Bastviken et al. 2008), which may be linked to energy input after ice-out (Wik et al. 2014), changes in the air pressure (Bastviken et al. 2004) and basin morphometry (Rasilo et al. 2015). During the summer stratification, formation of an anoxic hypolimnion typically results in high CH_4_ concentrations near the bottom due to favorable conditions for methanogenesis, and less favorable conditions for CH_4_ oxidation (Kankaala et al. 2007). However, this does not necessarily increase CH_4_ emissions to the atmosphere, because often a significant fraction of CH_4_ is oxidized in the overlying oxic water column before it enters the surface water (Bastviken et al. 2002; Kankaala et al. 2006; West et al. 2016).

Highest CH_4_ oxidation rates are detected near the oxycline (Rudd et al. 1974; Fallon et al. 1980; Kankaala et al. 2006; Bastviken et al. 2008), which can occur within the water column or at the sediment–water interface. In the oxycline, O_2_ is available as EA and CH_4_ as C and energy source (Rudd, Hamilton and Campbell 1974; Fallon et al. 1980). However, recent studies have also found anaerobic oxidation of methane (AOM) by anaerobic methane-oxidizing archaea (ANME) in sediments (Schubert et al. 2011) and in stratified water columns of freshwater lakes (Eller et al. 2005). While SO_4_^2−^-dependent AOM is an efficient CH_4_ sink in marine environments (Knittel and Boetius 2009), several EAs, such as NO_3_^−^, nitrite (NO_2_^−^), SO_4_^2−^, Fe^3+^ and manganese (Mn^4+^), have been demonstrated to be important drivers of AOM in freshwaters (Sivan et al. 2011; Deutzmann et al. 2014; Norði and Thamdrup 2014; Timmers et al. 2017). Nevertheless, the relevance of AOM in reducing CH_4_ emissions from freshwater lakes is still unclear and needs further research; e.g. Rissanen et al. (2017) did not detect AOM coupled to any of the inorganic alternative EAs in the sediments of two shallow boreal lakes in Finland, while significant AOM was observed in 13 out of 14 study lake sediments in the temperate, arctic and tropical zone (Martinez-Cruz et al. 2018).

Before the end of the century, the annual CH_4_ emissions from boreal lakes are projected to increase by 20–54% due to warming climate and longer ice-free seasons (Wik et al. 2016). Improved estimates of lacustrine CH_4_ dynamics are still required to forecast the future contributions of boreal lakes to the global CH_4_ budgets in a changing climate. Therefore, we applied stable isotope methods with ^13^C-labeled CH_4_, as well as measurements of natural abundance of ^13^C-CH_4_ and ^13^C-DIC (dissolved inorganic C), to reveal the controlling factors for CH_4_ production and oxidation in the water column of a typical seasonally O_2_-stratified boreal lake. Lake Kuivajärvi is a representative example of the numerous small brown-water lakes, that is located in a boreal landscape with managed coniferous forests and peatland and has high DOC concentrations (Miettinen et al. 2015). Previous work in Lake Kuivajärvi has focused on the lacustrine GHG fluxes, while the drivers behind these processes remain unknown. The objectives of this study were (1) to estimate CH_4_ production and oxidation during the development of summer stratification, and hypolimnetic hypoxia, and (2) to determine the environmental and biological factors that may explain CH_4_ oxidation in the water column. We hypothesized that the CH_4_ oxidation takes place in the hypolimnion, when O_2_ is below the detection limit of traditional O_2_ measurement techniques (hypoxia).

## Materials and methods

### Site description and measurements

Lake Kuivajärvi is a typical humic mesotrophic lake located in the boreal zone in central Finland (61° 50′ N, 24° 17′ E) close to the SMEAR II measuring station (Station for Measuring Ecosystem-Atmosphere Relations; Hari and Kulmala 2005). The lake, which has a northern and southern basin, has a surface area of 0.62 km^2^, length of 2.6 km and maximum depth of 13 m (Miettinen et al. 2015). The study area has mean annual temperature of 3.5 °C and precipitation of 711 mm (Pirinen et al. 2012). Each year the lake is frozen for approximately 5 months, and it is dimictic with complete turnover occurring immediately after ice-out and in the autumn (Heiskanen et al. 2015). The size of the catchment area is approximately 9.4 km^2^ and it consists of managed forests as well as peat- and agricultural land. For more information and e.g. bathymetric map of Lake Kuivajärvi, see Heiskanen et al. (2015). For total annual GHG fluxes as well as the timing of emissions from Lake Kuivajärvi, see Miettinen et al. (2015).

Water sampling was carried out four times between May and September in 2016 at the deepest point (13 m) of the southern basin of the lake. Sediment sampling was carried out in August. The sampling dates and measured variables (Table 1) were chosen to follow the development of the thermal stratification and the hypolimnetic hypoxia until the autumn turnover. The sampling was done on the measuring platform in the middle of the lake (Heiskanen et al. 2015). Data for weather conditions were obtained from the measuring station of Finnish Meteorological Institute (FMI) close to the SMEAR II station (Fig. S1; available at https://en.ilmatieteenlaitos.fi/open-data).

**Table 1:** Sampling schedule and the measured variables during each sampling of this study in 2016

Sampling date	Measured variables
25 May	Temperature, pH, the concentrations of O_2_, CH_4_ and CO_2_
18 July	Temperature, pH, the concentrations of O_2_, CH_4_, CO_2_, NO_x_^−^, NH_4_^+^ and SO_4_^2−^, δ^13^C-CH_4_, δ^13^C-DIC
15 August	Temperature, pH, the concentrations of O_2_, CH_4_, CO_2_, NO_x_^−^, NH_4_^+^, SO_4_^2−^ and DOC, δ^13^C-CH_4_, δ^13^C-DIC, ^13^C-CH_4_ oxidation experiment
5 September	Temperature, pH, the concentrations of O_2_, CH_4_, CO_2_, NO_x_^−^, NH_4_^+^, Fe, SO_4_^2−^, S_2_^−^ and DOC, δ^13^C-CH_4_, δ^13^C-DIC, ^13^C-CH_4_ oxidation experiment

### O_2_ concentration, water temperature and pH measurements

Vertical profiles of dissolved O_2_ concentration (mg l^−1^), O_2_ saturation (%) and water temperature (°C) were measured manually with a field meter YSI ProODO Optical Dissolved Oxygen Instrument (Yellow Springs Instruments, Yellow Springs, OH, USA; accuracy ± 0.2 °C, ± 0.1 mg O_2_ l^−1^ or ± 1% of reading). The measurements were done at 0.5 m intervals, starting from the surface water and continuing close to the bottom (12 m) without disturbing the sediment. The pH was measured in situ from samples taken with Limnos water sampler (length 30 cm, volume 2.0 dm^3^) at 1 m intervals using WTW ProfiLine pH 3110 (Xylem Inc., Weilheim, Germany).

### Nutrient and DOC analyses

Samples for nutrient and DOC analyses were collected at 1 m intervals from the surface water close to the bottom (11.5–11.75 m) by using Limnos water sampler. The samples were filtered through a plankton net (mesh size 25 μm) and a filter unit (pore size 0.22 μm, Millipore®, Sterivex, Darmstadt, Germany). The samples for nutrient analyses were stored frozen (−18 °C) until the further analysis with Ion Chromatograph (Dionex DX-120; Thermo Co., Bremen, Germany) for the SO_4_^2−^ concentrations, and colorimetric analysis for the NO_2_^−^ + NO_3_^−^ (NO_x_^−^; Miranda et al. 2001) and NH_4_^+^ concentrations (Fawcett and Scott 1960). The samples for DOC analyses were stored at +4 °C until analysis with a standard method (SFS-EN 1484), using Shimadzu TOC-V_CPH_ (Shimadzu Corp., Kyoto, Japan). The concentrations of total iron (Tot Fe)/ferrous iron (Fe^2+^) (the depths of 0–11.5 m) and sulphide (S_2_^−^) (the depths of 8–11.5 m) were determined with LCK320 and LCK653 cuvette test reagents, respectively, using Hach Lange DR2800 spectrophotometer (Hach Co., Loveland, CO, USA).

### The concentrations of CH_4_ and CO_2_ and stable isotopic analyses

The samples for the concentrations of CH_4_ and CO_2_ and stable isotopic analyses of CH_4_ were collected at 1 m intervals from the surface water close to the bottom (11.5–11.75 m) by using Limnos water sampler and processed as described in Miettinen et al. (2015). The CH_4_ and CO_2_ concentrations were measured using Agilent 7890B Gas Chromatograph (Agilent Technologies, Palo Alto, CA, USA) equipped with Gilson liquid handler GX271 autosampler (Gilson Inc., Middleton, WI, USA). The concentrations of CH_4_ and CO_2_ were calculated based on a one-point calibration with standard gas (AGA, Lidingö, Sweden), using Henry’s Law and the appropriate temperature relationships (Stumm and Morgan 1981). The δ^13^C-CH_4_ stable isotopes were analysed with Isoprime100 IRMS (Elementar UK Ltd., Cheadle, UK) coupled to an Isoprime TraceGas pre-concentration unit and calibrated using a standard gas mixture with known isotopic value for CH_4_ (− 46.7 ‰).

Water samples for the natural abundance of δ^13^C-DIC were collected at 1 m intervals from the surface water close to the bottom (11.5–11.75 m) and 3 ml of sample was injected into pre-evacuated 12 ml Labco Exetainers® (over-pressure released before injection). Exetainers® contained 300 μl of H_3_PO_4_ (85%) to ensure the transformation of bicarbonate ions to CO_2_. The samples were then stored upside down at +4 °C until the analysis. The samples from July were analysed with Delta Plus XP GC-IRMS (Thermo Co., Bremen, Germany), and the samples from August and September were analysed with Isoprime100 IRMS. The δ^13^C-DIC measurements were calibrated according to Coplen et al. (2006). The isotope results are reported in δ units (‰) relative to the international Vienna Pee Dee Belemnite (VPDB) standard.

### ^13^CH_4_ incubation experiment

The samples for ^13^CH_4_ oxidation measurement were collected from the water column from depths chosen on the basis of vertical profiles of O_2_. In August, the samples were collected at 6 m (2.48 mg l^−1^ O_2_), 11.5 m (1.35 mg l^−1^ O_2_) and the sediment surface (0.59 mg l^−1^ O_2_), and in September at 8 m (1.72 mg l^−1^ O_2_), 10 m (0.59 mg l^−1^ O_2_) and 11.5 m (0.44 mg l^−1^ O_2_). The sample water was transferred from Limnos sampler to 12 ml Exetainers® without a headspace and allowed to overflow. In August, the sediment samples were collected from the sediment surface (top 1 cm) by using Limnos sediment sampler with a slicing system and mixed with water collected right above the sediment surface at 1:4 ratio (2.4 ml of sediment and 9.6 ml of water). After 12 h pre-incubation in the dark at +4 °C to remove any traces of O_2_ introduced during the sampling, 0.1 ml of ^13^CH_4_ trace gas mixture was injected to each sample and the vials were shaken vigorously, resulting in the estimated final concentration of 25 µmol l^−1^ CH_4_ in each vial. ^13^CH_4_ trace gas mixture contained 140 ml of N_2_ and 10 ml of 99% ^13^C-CH_4_ in a N_2_-flushed, O_2_-free glass bottle with NaOH powder to remove any contaminating CO_2_. The disappearance of the ^13^CH_4_ bubble with sample water was observed visually for each vial. In August, there were four replicates and two non-labeled background samples for each sampling depth and time point. In September, each sampling depth had two replicates for 0 h time point, six replicates for 8, 16 and 24 h time points, and one non-labeled background sample for each time point. The incubations at +4 °C were terminated at 8-h intervals (0, 8, 16 and 24 h) by injecting 3 ml of incubated sample into evacuated 12 ml Exetainers® (over-pressure released before injection) that had 300 μl of H_3_PO_4_ (85%) in the bottom. The samples were analyzed for ^13^C-DIC with Isoprime100 IRMS. The excess ^13^C-DIC was calculated from the difference between the background ^13^C-DIC and the measured ^13^C-DIC for each time point. The excess ^13^C-DIC concentrations of each sampling depth were then plotted against time, and the slope of the line was used to describe the potential CH_4_ oxidation rate (nmol l^−1^ day^−1^). Considering that the incubations were amended with ^13^C-CH_4_ above ambient levels (0.02–0.9 µmol l^−1^ in Lake Kuivajärvi), and that the proportion of CH_4_-C bound to the microbial biomass was not measured, these values represent a potential or conservative rate.

### Statistical analysis

Two-tailed Spearman correlations were calculated between the gas concentrations/stable isotope values and variables such as depth, O_2_, temperature, pH, NO_x_^−^, NH_4_^+^, Fe^3+^, SO_4_^2−^, and DOC. Spearman’s rank correlation coefficient was chosen based on the Kolmogorov–Smirnov and Shapiro–Wilk normality test results (non-parametric data). Furthermore, simple linear regression analysis was used to study the relationship between the excess ^13^C-DIC production and incubation time in the ^13^CH_4_ oxidation experiments. Statistical analyses were performed with IBM SPSS Statistics 23.

## Results

### Thermal stratification associated development of hypolimnetic hypoxia

**Fig. 1:**
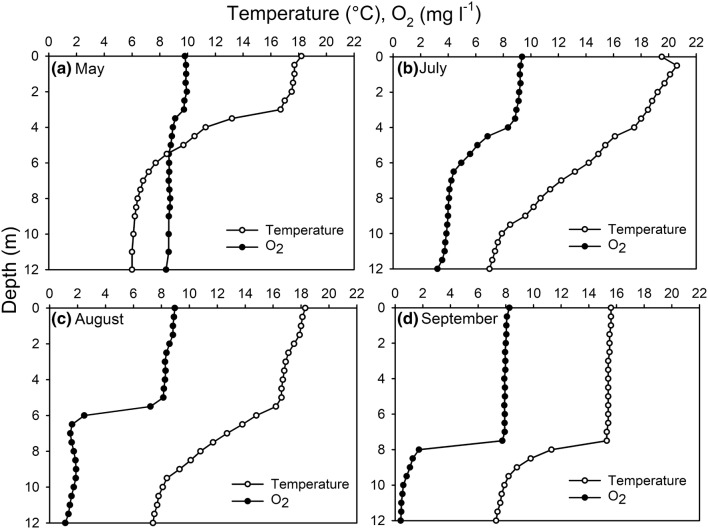
Depth profiles of water temperature (°C) and O_2_ concentration (mg l^−1^) in **a** May, **b** July, **c** August and **d** September

The depths of the warmer epilimnion and cooler hypolimnion were defined by assuming the metalimnion (thermocline) at the depth with a temperature change of more than 1 °C per meter. Water temperature in the epilimnion was highest in July (Fig. 1b) and lowest in September (Fig. 1d), while the hypolimnetic temperature was stable at about +6–7 °C throughout the study period. A thermocline varied in depth with changing seasons. In May, the thermal stratification was strongest, and temperature steeply decreased between 3 and 4 m (Fig. 1a), while in July there was no steep thermocline (Fig. 1b). In August, the temperature decreased after 5 m depth (Fig. 1c) and in September, there was a steep decrease of temperature at 8 m depth (Fig. 1d).

The whole water column was oxygenated in July (Fig. 1b), and the hypolimnetic hypoxia developed late in summer 2016. The oxycline ascended from the sediment to the water column during the development of summer stratification. Hypoxic conditions (< 2 mg l^−1^) were detected below 6 m depth in August (Fig. 1c), and below 8 m depth in the beginning of September (Fig. 1d).

### Depth profiles of water-quality variables

**Fig. 2:**
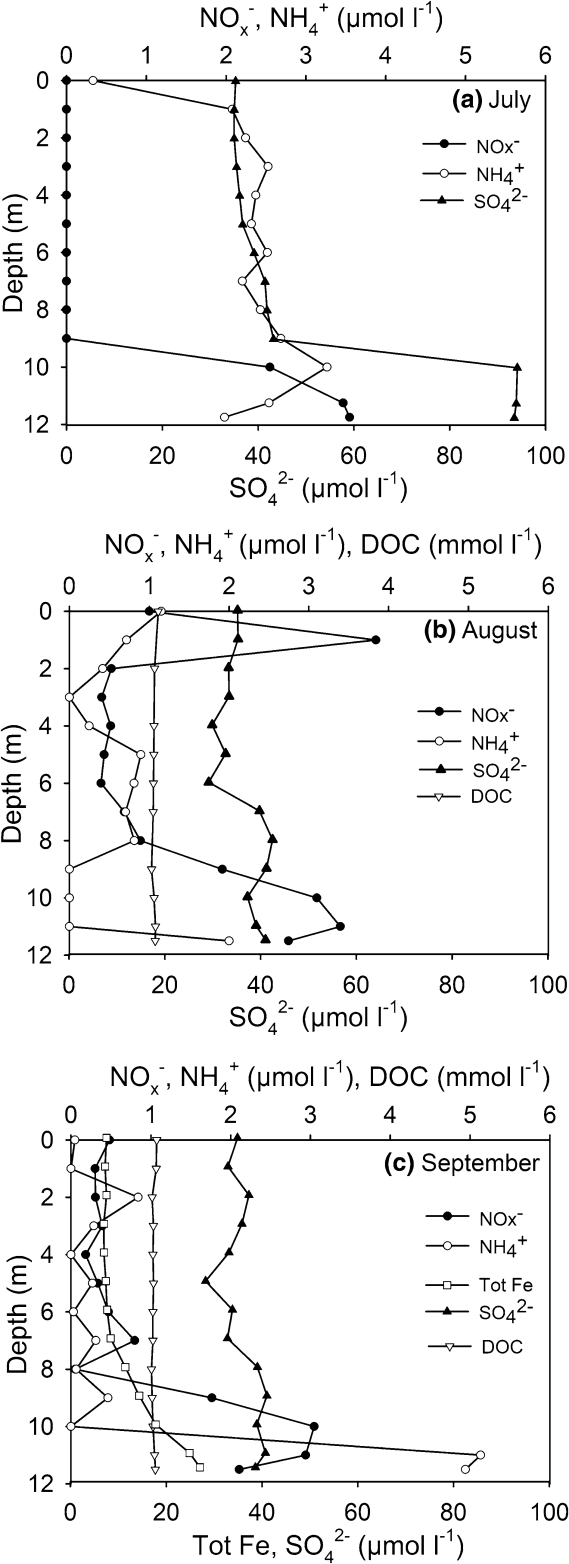
Concentrations of NO_x_^−^, NH_4_^+^ and SO_4_^2−^ in **a** July, **b** August and **c** September, concentrations of DOC in **b** August and **c** September, and concentrations of Tot Fe in **c** September. Note different scales on X-axis

In every sampling occasion, the NO_x_^−^ concentrations peaked in the hypolimnion (max. 3.5 µmol l^−1^), while the concentrations were mainly < 1 µmol l^−1^ at the depths of 0–8 m in August and September (Fig. 2b, c), and below the detection limit in July (Fig. 2a). The NH_4_^+^ concentrations remained mainly at < 2.5 µmol l^−1^, but in September, the hypolimnetic concentrations peaked to 5 µmol l^−1^ (Fig. 2c). In September, Total Fe concentrations slightly increased towards the hypolimnion (max. 27 µmol l^−1^; Fig. 2c). The SO_4_^2−^ concentrations stayed mainly between 30–45 µmol l^−1^ throughout the water column, except in July when the hypolimnetic concentrations of SO_4_^2−^ peaked to 94 µmol l^−1^ (Fig. 2a). In September, S_2_^−^ was not detected in the water column. DOC concentrations remained at < 1.1 mmol l^−1^, being highest in the epilimnion (Fig. 2b, c).

### Depth profiles of CH_4_ and CO_2_

**Fig. 3:**
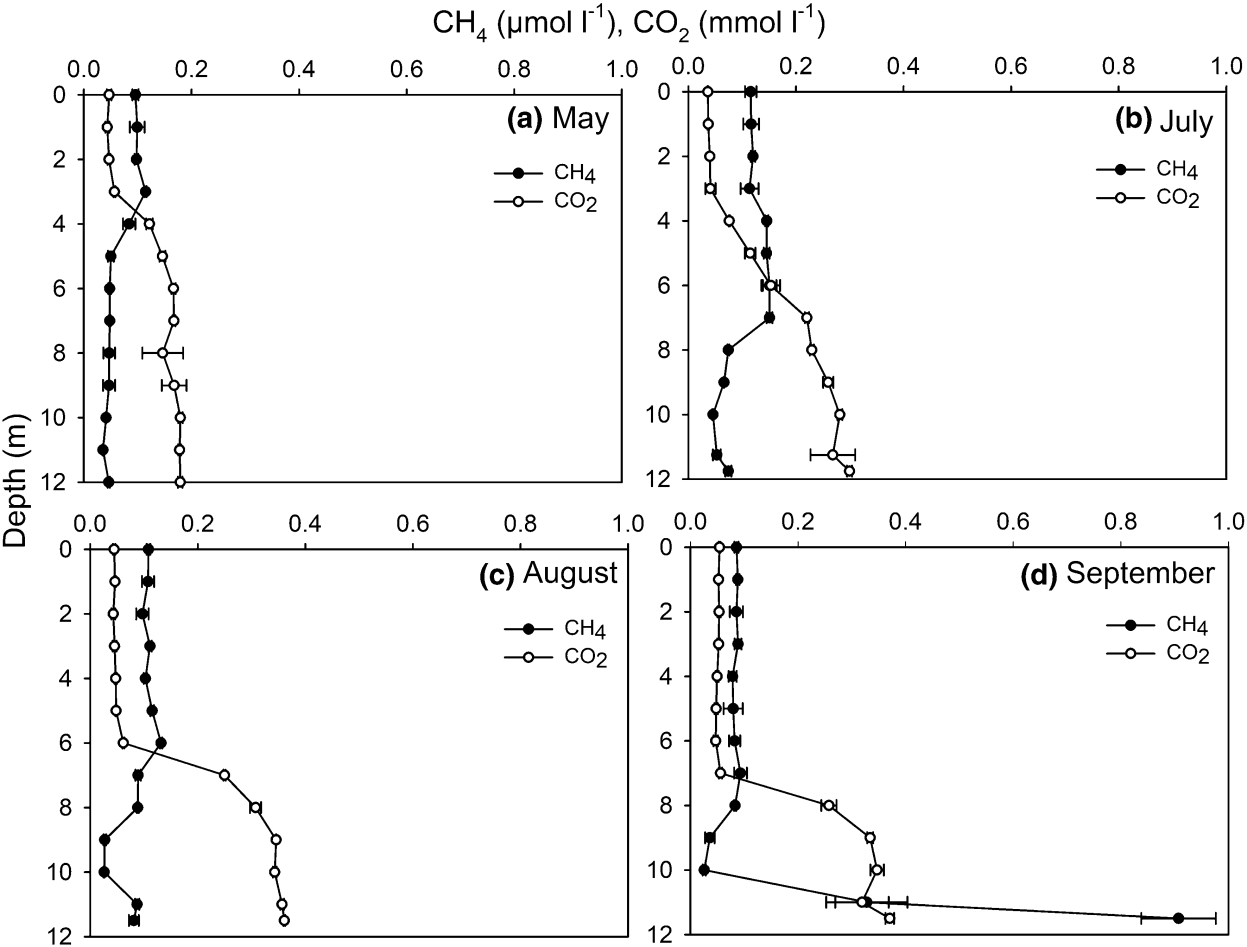
Depth profiles of CH_4_ and CO_2_ in **a** May, **b** July, **c** August and **d** September. Concentrations are presented as averages ± standard deviations (n = 2 or 3)

The epilimnetic CH_4_ concentrations were stable at approximately 0.1 μmol l^−1^ during the whole study period, while the concentrations in the metalimnion and hypolimnion changed seasonally. In early summer, the CH_4_ concentrations were highest in the upper water column; the water column maxima occurred at 3 m depth in May (0.115 ± 0.002 μmol l^−1^; Fig. 3a) and at 6 m depth in July and August (0.151 ± 0.013 μmol l^−1^ and 0.132 ± 0.002 μmol l^−1^; Fig. 3b, c). Below the peak, the CH_4_ concentrations started to decrease towards the bottom, until they slightly increased again at 11 m depth. In contrast, the CH_4_ concentrations in September were relatively low in the epilimnion and metalimnion but peaked in the hypoxic hypolimnion (0.91 ± 0.07 μmol l^−1^; Fig. 3d). The CH_4_ concentration correlated positively with water temperature, pH and the NH_4_^+^ concentration, and negatively with the water column depth and the concentrations of NO_x_^−^ and SO_4_^2−^ (Table 2).

**Table 2:** Spearman’s rank correlations between the average gas concentrations (n = 52) and stable isotopic values (n = 39) of CH_4_ and CO_2_, water column depth, temperature, the O_2_ concentration, pH (n = 52), and the concentrations of DOC (n = 23), NO_x_^−^, NH_4_^+^, SO_4_^2−^ (n = 39) and Fe^3+^ (n = 13)

	CH_4_ −concentration	CO_2_ concentration	δ^13^C-CH_4_	δ^13^C-DIC
Depth	− 0.490**	0.884**	n.s	− 0.872**
Temperature	0.638**	− 0.839**	− 0.332*	0.927**
O_2_	n.s	− 0.776**	n.s	0.975**
pH	0.625**	− 0.773**	− 0.347*	0.813**
DOC	n.s	n.s	− 0.444*	n.s
NO_x_^−^	− 0.403*	0.519**	n.s	− 0.494**
NH_4_^+^	0.396*	n.s	n.s	n.s
SO_4_^2−^	− 0.330*	0.705**	0.351*	− 0.652**
Fe^3+^	n.s	0.863**	n.s	− 0.802**
CH_4_		− 0.462**	− 0.726**	n.s
CO_2_	− 0.462**		n.s	− 0.946**

^*^Correlation is significant at the p < 0.05 level (2-tailed)

^**^Correlation is significant at the p < 0.01 level (2-tailed)

There was a negative correlation between the CH_4_ and CO_2_ concentrations (Table 2), their depth profiles being reflections of each other’s, particularly in May (Fig. 3a), but also during other sampling months. The epilimnetic CO_2_ concentrations remained stable from May to September, while the CO_2_ concentrations in the hypolimnion clearly increased from spring (0.179 ± 0.004 mmol l^−1^; Fig. 3a) to autumn (0.370 ± 0.008 mmol l^−1^; Fig. 3d). In May and July, the CO_2_ concentrations started to increase below 3 m depth (Fig. 3a, b), whereas in August and September, the CO_2_ concentrations increased simultaneously with the decreasing O_2_ concentrations (Fig. 3c, d). Throughout the sampling period, the CO_2_ concentration correlated negatively with water temperature, pH and the O_2_ concentration. There was also a positive correlation for CO_2_ with the water column depth, NO_x_^−^ and SO_4_^2−^. In addition, the CO_2_ and Fe^3+^ concentrations correlated positively in September (Table 2).

### Depth profiles of δ^13^C-CH_4_ and δ^13^C-DIC

**Fig. 4:**
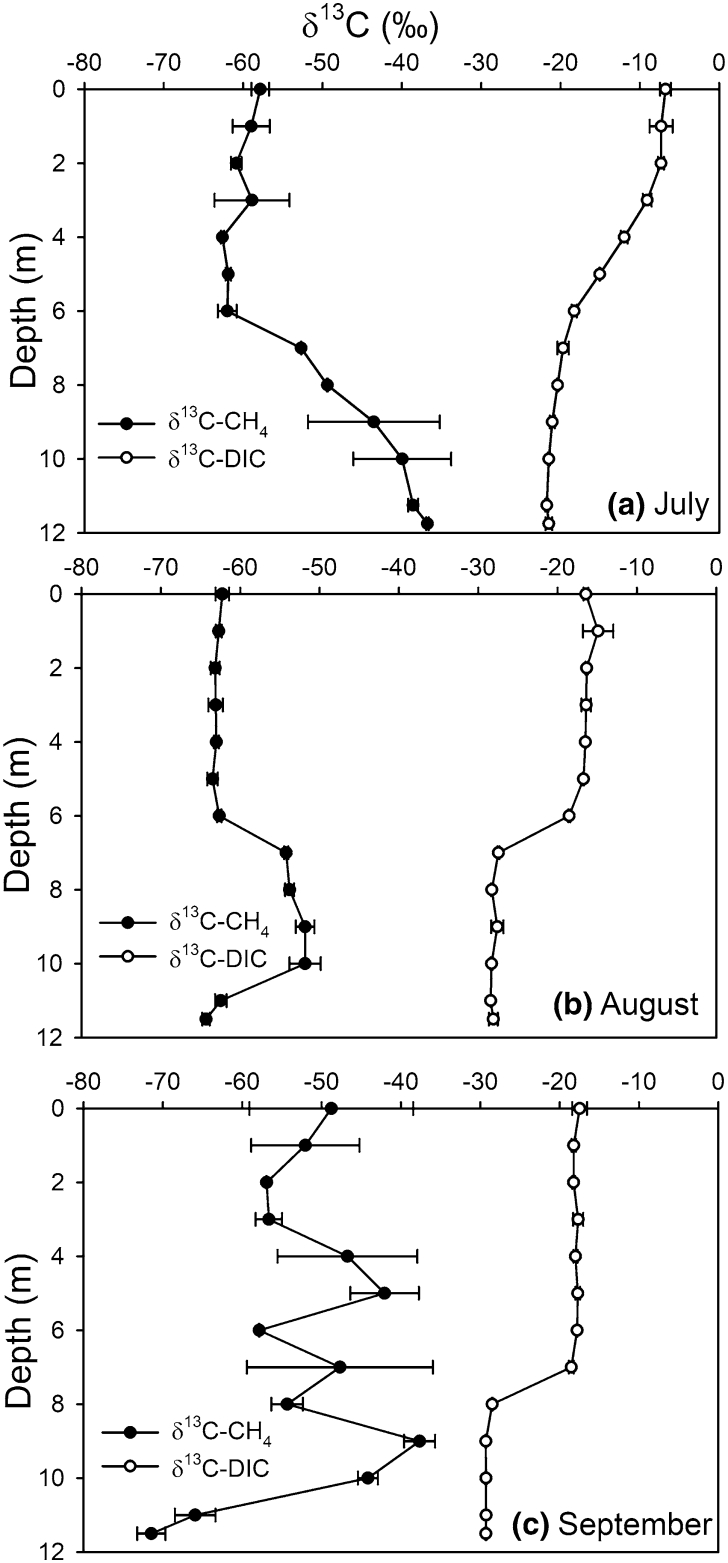
Depth profiles of δ^13^C-CH_4_ and δ^13^C-DIC (‰) in **a** July, **b** August and **c** September. Values are presented as averages ± standard deviations (n = 2 or 3)

There was a substantial temporal variation in the depth profiles of δ^13^C-CH_4_. In the hypolimnion, δ^13^C-CH_4_ decreased from − 36.8 ± 0.2 ‰ in July (Fig. 4a) to − 71.5 ± 1.8 ‰ in September (Fig. 4c). In August and September, the maximum δ^13^C-CH_4_ values were detected close to the oxycline at 9 m depth (− 51.8 ± 1.2 ‰ and − 37.6 ± 2.0 ‰, respectively). There was a significant negative correlation for the δ^13^C-CH_4_ with water temperature, pH, DOC concentration and CH_4_ concentration, while the δ^13^C-CH_4_ correlated positively with the SO_4_^2−^ concentration (Table 2).

Similarly to the profiles of CO_2_ and CH_4_, the depth profiles of δ^13^C-CH_4_ and δ^13^C-DIC were reflections of each other, and the δ^13^C-DIC values generally decreased from summer to autumn. The δ^13^C-DIC ranged from − 6.8 ± 0.7 to − 17.5 ± 0.9 ‰ in the epilimnion, and from − 21.5 ± 0.4 to − 29.3 ± 0.1 ‰ in the hypolimnion. In August and September, a notable decrease of δ^13^C-DIC occurred at the depths of 6–7 m (Fig. 4b, c), simultaneously with sudden O_2_ depletion, while in July the decrease of δ^13^C-DIC towards the bottom was more stable (Fig. 4a). The δ^13^C-DIC values correlated positively with water temperature, the O_2_ concentration and pH, whereas the δ^13^C-DIC correlated negatively with water column depth and the concentrations of NO_x_^−^, Fe^3+^, SO_4_^2−^ and CO_2_ (Table 2).

### The extent and potential rates of CH_4_ oxidation

In August, the estimated fraction of CH_4_ oxidized in the water column was 34% (calculated from the difference between δ^13^C-CH_4_ at the bottom and the maximum value of δ^13^C-CH_4_ at 9 m, as described in Kankaala et al. 2007). In September, the corresponding proportion was 91%.

**Fig. 5:**
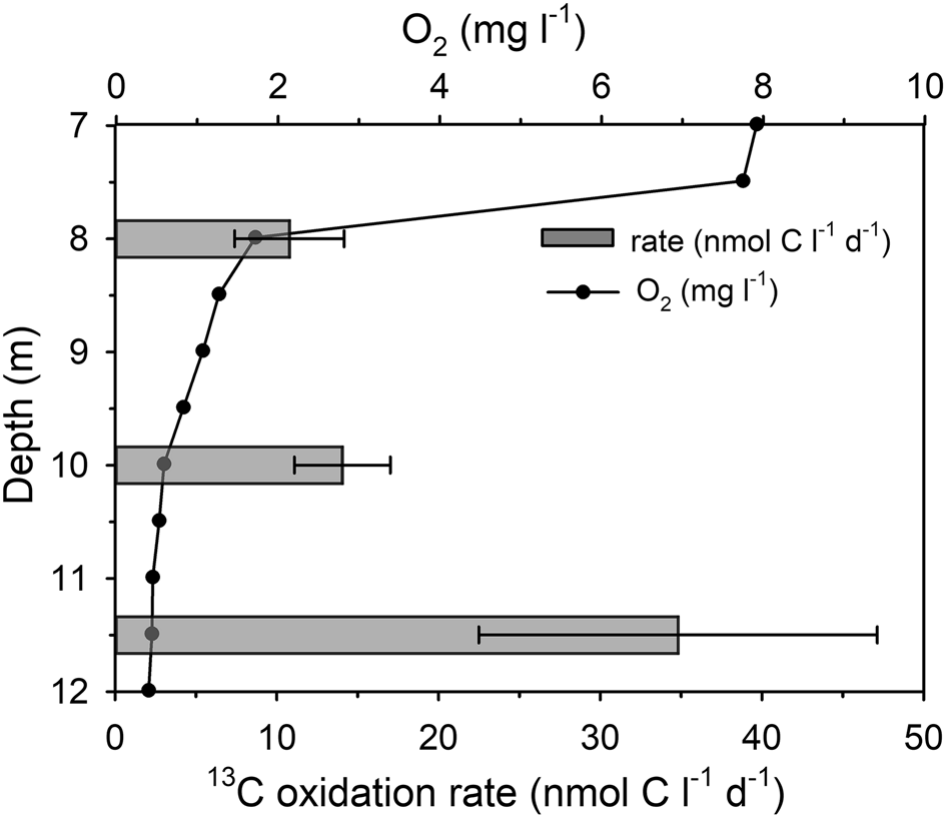
The O_2_ concentration (mg l^−1^) and the potential CH_4_ oxidation rates (nmol C l^−1^ d^−1^) ± standard errors determined with ^13^C-CH_4_-tracer in September (n = 18 at the depths of 8 and 11.5 m, and n = 17 at 10 m depth)

Potential CH_4_ oxidation was detected in September. Potential CH_4_ oxidation rates increased with depth from 10.8 ± 3.4 nmol l^−1^ day^−1^ at 8 m (p < 0.006**) to 34.8 ± 12.3 nmol l^−1^ day^−1^ at 11.5 m (p < 0.012*; Fig. 5). In contrast, the results from August did not show clear evidence of CH_4_ oxidation, since the tracer addition did not cause significant linear increase with time in samples from 6 m depth (p > 0.134) and sediment surface (p > 0.113). At 11.5 m depth, the values (Atom%) of labeled samples increased linearly with time (p > 0.349), but the large variation between replicates complicated interpretation of results and thus, CH_4_ oxidation during August cannot be confirmed (Fig. S2).

## Discussion

### The vertical distribution of CH_4_ in the water column

The epilimnetic concentrations of CH_4_ were similar to those previously recorded in Lake Kuivajärvi (Miettinen et al. 2015) and in Finnish lakes in general (e.g. 207 lakes studied by Juutinen et al. 2009). The hypolimnetic CH_4_ concentrations, however, were relatively low, even in September, when the hypolimnetic hypoxia created favorable conditions for methanogenesis (Capone and Kiene 1988). The low concentrations of CH_4_ were probably caused by the well-oxygenated water column in early summer.

From May to August, the highest concentrations of CH_4_ occurred in the upper water layers and the lowest concentrations in the hypolimnion. Even though the CH_4_ concentrations are expected to decrease in the well-oxygenated water column due to the methanotrophic activity (Kankaala et al. 2006; Bastviken et al. 2008), the lateral transport of CH_4_ from the littoral zone or surrounding peatlands (Murase et al. 2003; Ojala et al. 2011; Lopéz Bellido et al. 2013; Miettinen et al. 2015), as well as a rapid vertical release of CH_4_ from the sediment to the surface layers by ebullition (McGinnis et al. 2006), could cause such profiles. Also, internal lake oscillations might have contributed in vertical transfer of CH_4_ to the upper layers (Heiskanen et al. 2014; Stepanenko et al. 2016). Since there were no extreme rain events during the sampling periods to enable efficient lateral transport, an internal source for CH_4_ in the oxic water column seems more likely.

Although the CH_4_ concentrations did not correlate with the O_2_ conditions, simultaneous changes in the depth profiles of CH_4_ and nutrients (i.e. NO_x_^−^, NH_4_^+^ and SO_4_^2−^; Table 2) suggest that the availability of O_2_ was a major factor regulating both CH_4_ and nutrient concentrations in the water column. In the summer, well-oxygenated water column created favorable conditions for aerobic nitrification and oxidation of S_2_^−^ to SO_4_^2−^, while these conditions were less favorable for methanogenesis. Conversely, hypolimnetic hypoxia in September probably stimulated methanogens and ammonium-producing microbes simultaneously with denitrifying and sulphate-reducing bacteria.

The measured δ^13^C-CH_4_ values agreed with previous studies in boreal lakes (e.g. Bastviken et al. 2002, 2008; Kankaala et al. 2007). The δ^13^C-CH_4_ decreased with increases in CH_4_ concentration. Also, increases in temperature and DOC were associated with decreasing δ^13^C-CH_4_ values, as they are the key controlling factors for methanogenesis (Table 2; Bastviken et al. 2004, 2008; Duc et al. 2010). In August and September, CH_4_ production at the lake bottom was visible from the δ^13^C-CH_4_ profiles. The hypolimnetic decrease of δ^13^C-CH_4_ was substantial, particularly with maximum CH_4_ concentrations in September, which is consistent with biogenic CH_4_ being strongly ^13^C-depleted due to fractionation (Whiticar 1999). In July, ^13^C-enriched values of CH_4_ (− 37 ‰) at the lake bottom indicate that most of CH_4_ was formed and consumed within the sediment (Whiticar 1999).

Throughout the study period, the increases in CO_2_ with depth, simultaneously with decreasing δ^13^C-DIC and O_2_ concentration, indicate consumption of O_2_ and production of CO_2_ through in situ decomposition of OM in the hypolimnion (Miettinen et al. 2015). Furthermore, the decomposition of OM releases nutrients, such as NO_3_^−^ (McManus, Heinen and Baehr 2003), thus explaining the positive relationship between CO_2_ and NO_x_^−^.

In this study, we did not directly estimate the lake-atmosphere C gas exchange in Lake Kuivajärvi. However, the measured surface water CH_4_ and CO_2_ concentrations together with a 2-year (2011–2012) data set on atmospheric fluxes of C gases (Miettinen et al. 2015) confirm that Lake Kuivajärvi acts as a source of CH_4_ and CO_2_ to the atmosphere (the 2-year mean for CH_4_ approx. 0.06 mol m^–2^ y^–1^ and for CO_2_ 25.5 mol m^–2^ y^–1^).

### Water column CH_4_ oxidation and future perspectives in a changing climate

The transition of the active CH_4_ oxidation zone was clearly indicated by the δ^13^C-CH_4_ profiles. In July, CH_4_ remained ^13^C-enriched at the bottom, suggesting that CH_4_ was already oxidized in the sediment, because CH_4_ oxidation leaves a residual CH_4_ enriched in ^13^C (Whiticar 1999). During August and September, the most ^13^C-enriched values of CH_4_ were detected close to the oxycline, indicating the transition of CH_4_ oxidation from the sediment to the water column.

The estimated proportion of CH_4_ oxidized within the water column was 34% in August and 91% in September. The high efficiency of CH_4_ oxidation agrees well with previous studies, where the proportions of CH_4_ oxidized within the water column during summer stratification have ranged from 50 to 80% (Kankaala et al. 2006; Bastviken et al. 2008). Even though lakes generally represent an important natural source of atmospheric CH_4_, these results show that methanotrophic activity substantially reduces CH_4_ emissions from this typical, seasonally stratified lake.

In September, the potential CH_4_ oxidation rates gradually increased from the oxycline (8 m) to the hypoxic hypolimnion (11.5 m). Simultaneously, δ^13^C-CH_4_ strongly decreased, while δ^13^C-DIC remained stable. Although the highest CH_4_ oxidation rates are typically observed at the oxycline in the presence of O_2_ (Kankaala et al. 2006; Oswald et al. 2015), the maximum rates in the hypolimnion could be explained by the higher ambient concentration of CH_4_ (0.9 µmol l^−1^) at 11.5 m depth sustaining a larger population of methanotrophs (Sundh et al. 2005; Bastviken et al. 2008). However, the CH_4_ pool turnover time in September, calculated by dividing the CH_4_ concentration with the potential CH_4_ oxidation rate (e.g. Lin et al. 2005), was most rapid near the oxycline at the depths of 8–10 m (< 8 days), and slowest at 11.5 m (26 days).

When comparing the potential CH_4_ oxidation rates in Lake Kuivajärvi to other stratified systems (Milucka et al. 2015; Oswald et al. 2015, 2016), and assuming that the potential CH_4_ oxidation rate is proportional to the concentration of the added ^13^CH_4_ tracer, the maximum CH_4_ oxidation rates in Lake Kuivajärvi were approximately 5–8 times lower than in those lakes. Again, the higher ambient concentrations of CH_4_ (10–100-fold) in those systems most likely sustained a larger population of methanotrophs, thus leading to higher CH_4_ oxidation rates.

As noted in previous anoxic incubation studies (Blees et al. 2014; Norði and Thamdrup 2014; Rissanen et al. 2017), possibility of minor O_2_ contamination from the tracer injection cannot be excluded even with originally anoxic freshwater samples. Also, there might have been some residual O_2_ available for CH_4_ oxidation close to the detection limit of O_2_ sensor. Indeed, the maximum CH_4_ oxidation rates in the hypolimnion imply that episodic appearance of O_2_ (e.g. downwelling of oxygenated water) in otherwise hypoxic layers potentially fueled methanotrophy below the oxycline, thus stimulating microaerobic CH_4_ oxidation (Kalyuzhnaya et al. 2013; Blees et al. 2014; Kits et al. 2015). Recently, aerobic gamma-proteobacterial methanotrophs have been reported to almost exclusively dominate the methanotrophic community in both oxic and anoxic layers of boreal and temperate lakes (Milucka et al. 2015; Oswald et al. 2016; Rissanen et al. 2018). Further research identifying the microbial communities involved in these processes is required to confirm whether the metabolism of methane-oxidizing microbes in Lake Kuivajärvi was aerobic or anaerobic.

In the future, as the aquatic systems in the boreal zone are exposed to increasing terrestrial organic C load due to climate-induced changes in precipitation and air temperature (Lepistö et al. 2008; Sarkkola et al. 2009; Couture et al. 2012; Pumpanen et al. 2014; Kiuru et al. 2018), the accelerated decomposition of OM might emphasize the role of alternative inorganic EAs in CH_4_ oxidation. The development of summer stratification, on the other hand, suggests that the annual CH_4_ emissions will remain largely regulated by aerobic CH_4_ consumption due to the well-oxygenated water column throughout the summer.

### Potential effects of warming climate on the onset of thermal stratification and hypolimnetic hypoxia

As a consequence of warm spring, Lake Kuivajärvi began to thermally stratify soon after ice-out and rapidly formed a warm epilimnetic layer, while the bottom waters remained cold and oxygenated. Long-term trends of thermal conditions have previously shown an extension of the summer stratification period in dimictic lakes of the boreal and temperate region (Gerten and Adrian 2002; Rösner et al. 2012; Magee and Wu 2017). Browning of boreal lakes together with warming climate causes earlier thermal stratification due to dark humic waters absorbing solar radiation (Heiskanen et al. 2015). Since the mean air temperatures during spring months from March to May have clearly increased in Finland (Mikkonen et al. 2015) and will continue to increase in the future (Ruosteenoja et al. 2016), this kind of early thermal stratification is becoming more common in boreal brown-water lakes (Heiskanen et al. 2015; Davidson et al. 2018; Kiuru et al. 2018; Mammarella et al. 2018). Since the hypolimnetic hypoxia did not begin until early autumn, it most likely lasted only few weeks before the autumn turnover. Previously, the duration of hypolimnetic hypoxia in Lake Kuivajärvi has varied from 3 weeks to more than 2 months (Miettinen et al. 2015), after which the autumn turnover has taken place in the beginning of October (e.g. Heiskanen et al. 2015). The results of this experiment represent the future O_2_ conditions in boreal lakes, showing that earlier thermal stratification with cold hypolimnion delays the period of hypolimnetic hypoxia and thus limits CH_4_ production.

## Conclusions

The zone of CH_4_ oxidation ascended from the sediment to the water column in the late phases of summer stratification, and our results showed that the CH_4_ oxidation potential was highest in the hypoxic hypolimnion. During hypolimnetic hypoxia, 91% of available CH_4_ was oxidized in the active CH_4_ oxidation zone, while 9% was potentially released to the atmosphere. Even though lakes represent an important natural source of atmospheric CH_4_ due to their large areal extent, our results demonstrate that earlier thermal stratification with cold, well-oxygenated hypolimnion will delay the period of hypolimnetic hypoxia, thus limiting CH_4_ production. Moreover, changes in the stratification dynamics of boreal lakes are expected due to the higher atmospheric temperatures and brownification. Therefore, the expected increase in the lacustrine CH_4_ emissions as a consequence of increasing organic C load from forested catchments may be partially counteracted by the later development of hypoxia.

## Electronic supplementary material

Below is the link to the electronic supplementary material.

Supplementary file1 (DOCX 540 kb)
