# Supplementary material for: CH4 oxidation in a boreal lake during the development of hypolimnetic hypoxia
Source: Aquat Sci. 2019 Dec 28;82(2):19. doi: 10.1007/s00027-019-0690-8 (PMC7181431; doi:10.1007/s00027-019-0690-8)
Supplement: Supplementary file 1 — Supplementary file1 (DOCX 540 kb) [file 27_2019_690_MOESM1_ESM.docx]

**Electronic supplementary material**

**CH_4_ oxidation in a boreal lake during the development of hypolimnetic hypoxia**

**Taija Saarela^1^, Antti J Rissanen^2^, Anne Ojala^3,4,5^, Jukka Pumpanen^1^, Sanni L Aalto^1^, Marja Tiirola^6^, Timo Vesala^7^, and Helena Jäntti^1^**

^1^ Department of Environmental and Biological Sciences, University of Eastern Finland, Yliopistonranta 1 E, FI-70210 Kuopio, Finland

^2^ Faculty of Engineering and Natural Sciences, Tampere University, Korkeakoulunkatu 6, FI-33720 Tampere, Finland

^3^ Ecosystems and Environment Research Programme, Faculty of Biological and Environmental Sciences, University of Helsinki, Viikinkaari 1, FI-00014 University of Helsinki, Helsinki, Finland

^4^ Institute of Atmospheric and Earth System Research (INAR)/Forest Sciences, Faculty of Agriculture and Forestry, University of Helsinki, Viikinkaari 1, FI-00014 University of Helsinki, Helsinki, Finland

^5^ Helsinki Institute of Sustainability Science (HELSUS), Faculty of Biological and Environmental Sciences, University of Helsinki, Viikinkaari 1, FI-00014 University of Helsinki, Helsinki, Finland

^6^ Department of Biological and Environmental Sciences, University of Jyväskylä, Survontie 9 C, FI-40014 Jyväskylä, Finland

^7^ Institute of Atmospheric and Earth System Research (INAR)/Physics, Faculty of Sciences, University of Helsinki, Gustaf Hällströmin katu 2, FI-00560 Helsinki, Finland

Corresponding Author:

Taija Saarela

Email address: taija.saarela@uef.fi

Telephone number: +358 50 512 9387

**
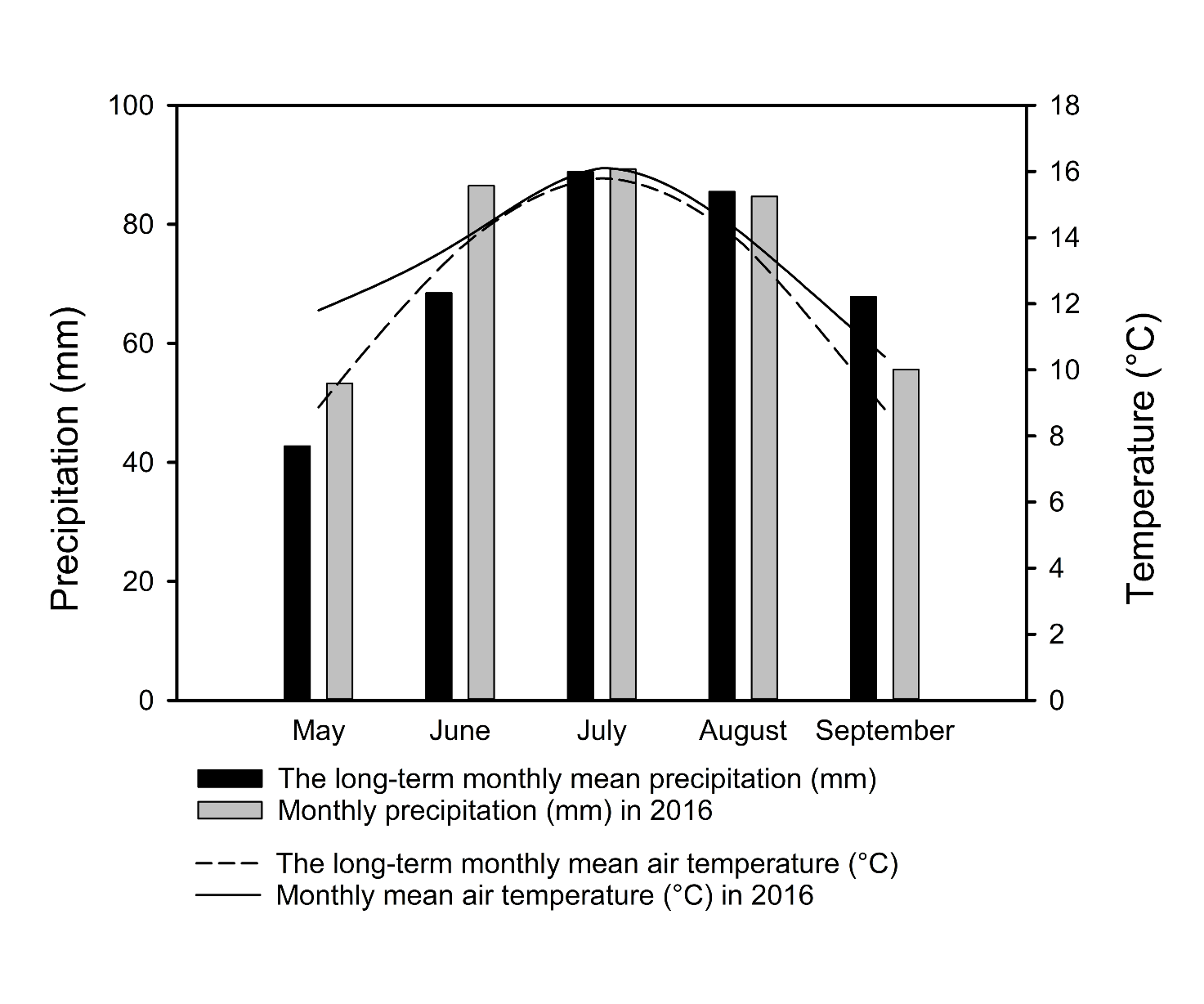
**

**Fig. S1** Monthly mean air temperature (°C) and precipitation (mm) from May to September 2016 in comparison to the long-term averages in 1959-2016. Data obtained from the measuring station of the Finnish Meteorological Institute (FMI) close to the SMEAR II station, Hyytiälä (Finnish Meteorological Institute 2016)

**
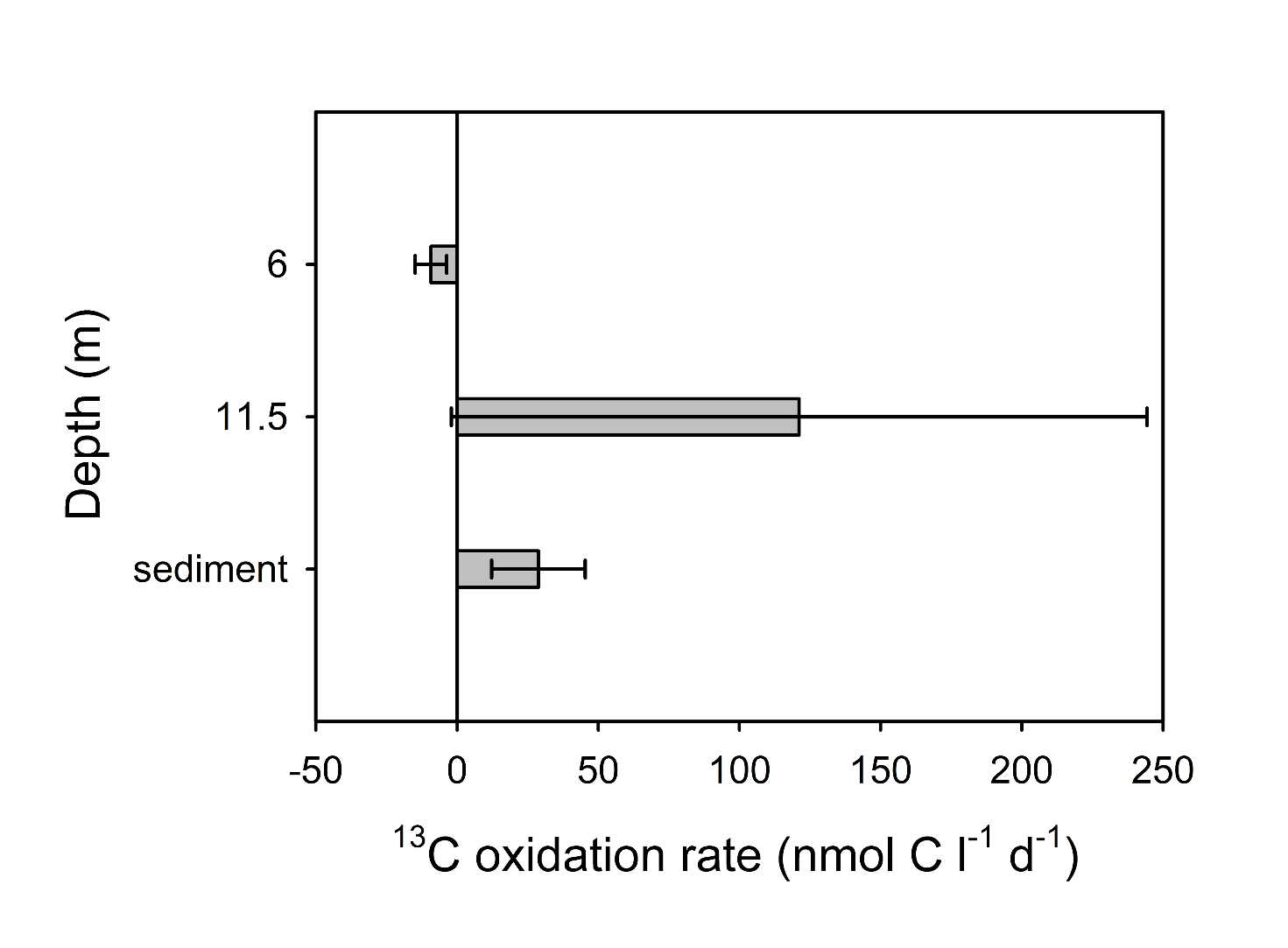
**

**Fig. S2** The potential CH_4_ oxidation rates (nmol C l^-1^ d^-1^) +/- standard errors determined with ^13^C-CH_4_-tracer in August (n=10 at 6 m depth, and n=12 at 11.5 m depth and sediment surface)
